# Evaluation of a Decision Aid in Kidney Care Counseling for Older Adults

**DOI:** 10.1016/j.ekir.2026.106371

**Published:** 2026-02-25

**Authors:** Semra Ozdemir, Lina Hui Lin Choong, Boon Wee Teo, Shien Wen Sheryl Gan, Vinh Anh Huynh, Eric Andrew Finkelstein, myKIDNEY study group, Jason Chon Jun Choo, Tazeen Jafar, Gek Cher Chan, Guozhang Lee, Lydia Wei Wei Lim

**Affiliations:** 1 Lien Center for Palliative Care, Duke-NUS Medical School, Singapore; 2 Department of Population Health Sciences, School of Medicine, Duke Clinical Research Institute, Duke University, Durham, NC, USA; 3 Department of Renal Medicine, Singapore General Hospital, Singapore; 4 Division of Nephrology, Department of Medicine, National University Hospital, Singapore; 5 Saw Swee Hock School of Public Health, National University of Singapore, Singapore

**Keywords:** counseling, decision aid, end-stage kidney disease, older patients

## Abstract

**Introduction:**

We evaluated the effectiveness of a web-based patient decision aid (PDA) developed for older adults with end-stage kidney disease (ESKD) and their caregivers, integrated into kidney care counseling.

**Methods:**

A pragmatic pre- and post-trial with a comparison group was conducted in Singapore with patients aged ≥ 70 years with ESKD and their caregivers. Usual care participants received standard counseling, whereas intervention arm received counseling using the PDA. The primary outcome was change in decisional conflict from pre- to post-counseling between arms. Secondary outcomes were conservative management (CM) awareness, stated treatment preference at postcounseling, and real-life treatment choice at 6 months. Analyses used a generalized linear mixed model for the primary outcome and logistic regressions for secondary outcomes.

**Results:**

A total of 143 participants were enrolled (75 patients and 68 caregivers). Caregivers in the intervention arm experienced significantly greater reductions in decisional conflict compared with usual care (β = -4.48; *P* < 0.001), whereas reductions did not differ between arms for the patients (β = 0.05; *P* = 0.97). Postcounseling, patient CM awareness increased more in the intervention arm (82.6% vs. 69.2%; odds ratio (OR) = 7.24; *P* < 0.01). Patients receiving the PDA were less likely to prefer dialysis postcounseling (OR = 0.33; *P* = 0.12) and were less likely to initiate dialysis within 6 months (OR = 0.21; *P* = 0.049).

**Conclusion:**

Although future studies are needed to confirm these results, this study provides preliminary, suggestive evidence that integrating a web-based PDA into kidney care counseling may reduce caregiver decisional conflict, improve patient awareness of CM, and shift both stated and actual treatment choices away from dialysis.

## Graphical Abstract

**Figure:**
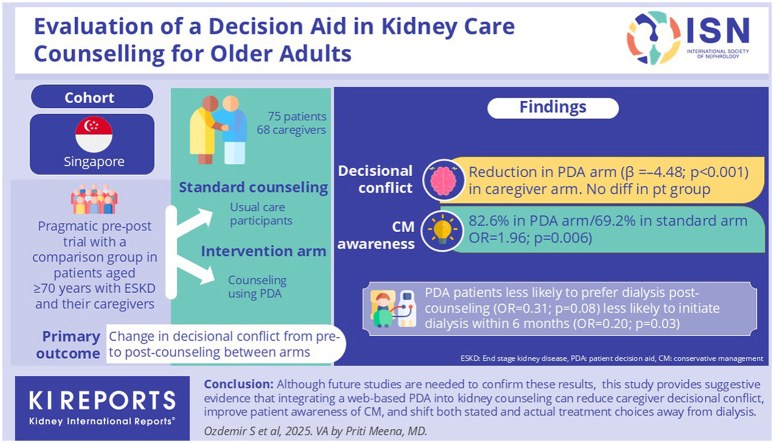

Because of aging populations and rising rates of chronic disease, the incidence of ESKD is increasing among older adults.^1^^,^^2^ Dialysis is often the first-line treatment for ESKD.^3^ However, its benefits for older adults with multiple comorbidities are often limited.^4^, ^5^, ^6^, ^7^ For those aged 75 years and older, dialysis offers minimal survival advantage,^8^, ^9^, ^10^ is associated with increased hospitalization rates,^9^^,^^11^ and confers higher disease burden compared with CM, which focuses solely on symptom control. Older adults on dialysis are also less likely to access palliative care services^9^ and more likely to die in a hospital than those receiving CM.^12^ For these reasons, the Kidney Disease: Improving Global Outcomes group^13^ and the American Society of Nephrology^14^ advocate for greater education and shared decision-making to ensure that patients consider all viable options, including various modes of dialysis and CM, before making a treatment choice.

Despite these recommendations, many older adults do not engage in a shared decision-making process when making ESKD treatment decisions.^15^ The majority choose dialysis with only a limited understanding of its costs, survival outcomes, and impact on quality of life, and how these compare to CM.^16^^,^^17^ Most are unaware that CM is a reasonable alternative or equate it to “doing nothing”^18^ or “immediate death”.^19^ This is partly because physicians often present dialysis as the default option.^20^ This lack of informed decision-making results in high rates of decisional regret.^10^^,^^21^^,^^22^ Up to 61% of patients who initiate dialysis later question their decision, especially if they felt pressured or inadequately informed.^10^^,^^21^

PDAs enhance shared decision-making by presenting balanced, evidence-based information about treatment options. Shared decision-making, regardless of treatment choice, leads to greater patient satisfaction with both the decision-making process and treatment outcomes,^23^ improves patient knowledge, increases engagement, and reduces decisional conflict.^24^, ^25^, ^26^, ^27^, ^28^ With these benefits in mind, we developed myKIDNEY, a PDA, which provides information about dialysis modalities and CM tailored for older patients and their family caregivers in Singapore. The PDA was initially created as a booklet^29^ and later adapted into a web-based format, following the International Patient Decision Aid Standard guidelines and in collaboration with an advisory panel of patients, caregivers, and clinicians. It was designed to support both patients and their family caregivers, given the significant role that family caregivers play in medical decision-making for older adults.^30^^,^^31^

This study presents findings from a pragmatic pre- and post-trial with a comparison arm evaluating the effectiveness of integrating myKIDNEY into kidney care counseling sessions compared with standard counseling without the PDA (i.e., usual care). The primary outcome was the difference between the 2 study arms in the change in decisional conflict from pre- to post-counseling, measured using a decisional conflict scale (DCS). Secondary outcomes included the following: (i) awareness of CM, (ii) stated preference for CM versus dialysis post counseling, and (iii) actual treatment choice, as documented in electronic health records 6 months after counseling. We hypothesized that participants who receive counseling with myKIDNEY would report lower decisional conflict compared with those receiving usual care. We also hypothesized that a larger proportion of those in the PDA arm would be aware of CM, and, as a result, a smaller proportion would choose dialysis as their preferred treatment pathway postcounseling and would be less likely to initiate dialysis within 6 months after counseling.

## Methods

### Study Setting and Design

The myKIDNEY study (ClinicalTrials.gov, NCT05407896) was conducted at the renal medicine clinics at 2 of the largest public hospitals in Singapore, the Singapore General Hospital and the National University Hospital. Before the introduction of myKIDNEY, the study sites offered counseling sessions for patients with a diagnosis of incident CKD Stage 5 with glomerular filteration rate < 15 ml/min. However, these sessions provided limited information about CM and the materials were not tailored to the needs of older patients. As a result, less than 10% of patients presenting with a diagnosis of ESKD elect to receive CM. Approximately 86% of patients without a plan for ESKD started dialysis urgently with a dialysis catheter, which is associated with increased hospitalizations and worse clinical outcomes.^32^

A pragmatic pre- and post-design was selected because of practical and logistical constraints. A cluster randomized trial was not feasible because of the limited number of available sites, and individual-level randomization posed a high risk of contamination as only a few kidney counselors were available to support intervention delivery. Additionally, once the PDA became available, hospital partners expressed discomfort with withholding it from eligible patients, further precluding randomization. Given these considerations and our assessment that time-related confounding would likely be minimal, we adopted a pre- and post-design approach. Patients were enrolled in the usual care arm while the web-based PDA was being developed. Recruitment for the intervention arm commenced after the usual care arm reached its target sample size and the PDA was ready for use.

Usual care recruitment took place from July 2021 to September 2022. Intervention arm recruitment occurred from December 2023 to September 2024. For both arms, the study coordinator intercepted patients and caregivers who were scheduled for a kidney care counseling session for dialysis consideration. Six months post counseling, participants’ actual treatment choices were extracted from their medical records.

### Study Participants

The inclusion criteria for patients were a diagnosis of incident CKD Stage 5 with glomerular filteration rate < 15 ml/min within the last 6 months, age 70 years or older, no previous kidney counseling, able to speak English or Mandarin, and cognitively intact as determined by the Abbreviated Mental Test. The exclusion criteria included being deemed mentally incompetent. The inclusion criteria for caregivers required them to be the primary informal caregiver of an eligible patient, aged 21 years or older, a direct relative of the patient, and actively involved in patient care and decision-making. Foreign domestic workers and minors were excluded.

Potential participants were identified from hospital electronic medical records. Contact information was then shared with a research coordinator who contacted potentially eligible patients either before their kidney care counseling appointment over the phone or approached them in person at the renal clinics on the day of their appointment to present study information. If a patient expressed interest, the study was further explained, and informed consent was obtained. Research coordinators then administered the Abbreviated Mental Test to determine the patient’s cognitive status, using the standard Abbreviated Mental Test guidelines that account for the patient’s age and years of education.

Eligible patients were asked if their caregiver might be interested in participating. If so, the caregiver was approached to assess eligibility and obtain informed consent. In cases where patients met the first 3 eligibility criteria (i.e., glomerular filteration rate < 15 ml/min, aged 70 years or older, and had not undergone kidney counseling with a kidney counselor) but were found to be cognitively impaired, not literate in English or Mandarin, or declined to participate, their caregivers were still invited to participate.

### Usual Care and Intervention Arms

In the usual care arm, participants received standard kidney care counseling using existing educational materials designed for patients of all ages. These materials primarily focused on comparing hemodialysis, peritoneal dialysis, and kidney transplant.

In the intervention arm, participants received counseling with the myKIDNEY web-based decision aid, developed in accordance with the International Patient Decision Aid Standards and the Ottawa Decision Aid Framework. The content of the decision aid arm was adapted from a previously developed brochure and video.^29^ These materials were then converted into an online, web-based tool with input from user design experts and were iteratively tested and refined based on interviews with 6 patients, 10 caregivers, and 10 healthcare providers who care for patients with ESKD. The PDA included the following sections: (i) information about ESKD, (ii) overview of treatment options (hemodialysis, peritoneal dialysis, and CM), (iii) side-by-side comparison of treatment options, (iv) personalized expected survival estimates based on age and comorbidities, (v) guidance on how to choose a treatment, (vi) values clarification exercises, (vii) tips for discussing concerns with loved ones and healthcare providers, (viii) information on changing treatment plans, (ix) advice for caregivers, and (x) videos providing key information about treatment options and showing patient and caregiver testimonials.

In Singapore, individuals must be below the age of 70 to be placed on the national waiting list for a transplant. Although patients above 70 remain eligible for living donor transplants, this is extremely rare. For this reason, in consultation with the clinician members of our team, we decided not to include kidney transplantation as one of the options presented in the decision aid.

In the intervention arm, counselors guided participants through the PDA, summarized key information, provided quick response codes, and encouraged participants to watch the video testimonials at a later time. They also facilitated the values clarification exercise which used a best-worst scaling method.^33^ This exercise included a series of questions where participants chose the most and least important factors when considering management of their kidney failure across a range of different factors. Based on responses, participants are presented with a ranking of these factors and how their priorities align with a particular treatment (dialysis or CM).

In both arms, when caregivers provided consent, both the patient and caregiver participated in the counseling session. Counseling sessions in both arms were scheduled for 45 minutes. The counselors underwent standardized training before delivering study sessions. Training for the usual care and intervention arms was conducted separately to avoid cross-contamination; however, within each arm, counselors received identical training. The training emphasized protocol adherence and consistent communication of key messages. For the intervention arm, counselors also received hands-on training in navigation and use of the decision aid, including guidance on presenting survival estimates and facilitating the values clarification exercise. These procedures were implemented to minimize inter-counselor variability in session delivery.

All study participants, including caregivers, completed a pre- and post-counseling survey. Depending on their preference, participants either completed the online surveys independently on tablets or with assistance from a research coordinator. Each participant received a SGD100 (∼$75) voucher as reimbursement for their participation.

### Outcomes

The primary effectiveness outcome was the difference between the 2 arms in the change in decisional conflict pre- and post-counseling. Decisional conflict is defined as the uncertainty individuals experience when choosing among healthcare options, typically arising from limited knowledge, unclear values, or inadequate support.^34^ Higher decisional conflict has been linked to delayed or poorly informed decision-making, lower confidence in chosen treatments, and greater decision regret.^35^^,^^36^ Decisional conflict was measured by the DCS, a validated scale comprising 16 items with 5 response options, scores ranging from 0 to 100.^34^^,^^37^ Higher scores indicate greater levels of decisional conflict.

Secondary outcomes were as follows: (i) being aware of CM at postcounseling, (ii) stated preference for CM versus dialysis at postcounseling, and (iii) actual treatment choice documented in the electronic health record at 6 months. Those initially unsure about their treatment preference post counseling were followed up via phone within 1 week of the counseling session to obtain a preference.

### Sample Size

A small to medium effect size based on Cohen’s *d* is roughly 0.30 to 0.40, which corresponds to a change of about 5.5 to 8 points on the DCS, assuming a standard deviation of 18. Therefore, we powered the study to detect a 7-point difference with this standard deviation. This required a sample size of 104 participants per arm (208 in total), assuming 80% power and 1-sided 5% type 1 error rate.

### Statistical Analysis

Analysis was conducted using an intention-to-treat approach. For outcomes measured both pre- and post-counseling, DCS score and CM awareness, we used mixed-effects regression models with participant-level random intercepts to account for within-participant correlation. Specifically, DCS scores (continuous outcomes) were analyzed using mixed-effects linear regression models (Gaussian family, identity link) with an exchangeable covariance structure (STATA mixed command), and awareness of CM treatment (binary outcome) was analyzed using mixed-effects logistic regression models (binomial family and logit link) with the same exchangeable covariance structure and random-effects specification (STATA melogit command). These models estimated the fixed effect of the intervention while accounting for repeated measurements within participants.

For postcounseling treatment preferences (dialysis vs. nondialysis and CM vs nonCM) and actual treatment choice at 6 months, we used logistic regression models (generalized linear models with a binomial distribution and logit link function). Adjusted models controlled for baseline differences in the relevant outcome, age, sex, education level, presence of at least 1 comorbidity, and health status measured by the Kidney Disease Quality of Life Instrument-36 (patient sample only).^38^ All analyses were conducted on complete cases using STATA (version 16.0, StataCorp LLC., Texas) and conservatively reported using 2-sided confidence intervals (CIs) and *P* values.

## Results

### Participant Characteristics

A total of 143 participants were enrolled, 106 in the usual care arm (52 patients and 54 caregivers), but because of recruitment challenges, only 37 were included in the intervention arm (23 patients and 14 caregivers). Table 1 presents the baseline characteristics. Among the patients, the mean age was 76.7 ± 4.7 years. The majority were male (61%), Chinese (81%), married (71%), had at least a secondary education (67%), and had diabetes (61%). Only 15% were working full or part-time. Among caregivers, the mean age was 55.2 ± 12.2 years. Roughly three-fourths of the caregivers were adult children of the patients. No statistically significant differences were observed across arms.

**Table 1:** Descriptive statistics (*N* = 143)

	Patients (*n* = 75)			Caregivers (*n* = 68)		
	Usual care	Intervention	*P* value	Usual Care	Intervention	*P* value
	*n* = 52	*n* = 23		*n* = 54	*n* = 14	
Age, yr, mean (SD)	76.1 (4.6)	77.9 (4.7)	0.13	54.9 (12.8)	56.5 (9.9)	0.67
Sex			0.75			0.068
Female	20 (38.5%)	8 (34.8%)		35 (64.8%)	6 (42.9%)	
Male	31 (59.6%)	15 (65.2%)		19 (35.2%)	7 (50.0%)	
Ethnic group			0.28			0.092
Chinese	39 (75.0%)	22 (95.7%)		40 (74.1%)	13 (92.9%)	
Indian	4 (7.7%)	1 (4.3%)		4 (7.4%)	0 (0.0%)	
Malay	7 (13.5%)	0 (0.0%)		8 (14.8%)	0 (0.0%)	
Others	1 (1.9%)	0 (0.0%)		2 (3.7%)	0 (0.0%)	
Married	36 (69.2%)	17 (73.9%)	0.68	41 (75.9%)	9 (64.3%)	0.38
Education level^a^			0.88			0.056
Primary school and below	17 (32.7%)	8 (34.8%)		2 (3.7%)	0 (0.0%)	
Secondary/Vocational/ITE	29 (55.8%)	12 (52.2%)		22 (40.7%)	2 (14.3%)	
JC/Poly/University and above	5 (9.6%)	3 (13.0%)		30 (55.6%)	11 (78.6%)	
Working full-time or part-time	8 (15.4%)	3 (13.0%)	0.79	33 (61.1%)	11 (78.6%)	0.22
Household income			0.11			0.075
Less than SG$2,000	12 (23.1%)	6 (26.1%)		9 (16.7%)	1 (7.1%)	
SG$2,000 to less than SG$5,000	1 (1.9%)	3 (13.0%)		6 (11.1%)	2 (14.3%)	
SG$5,000 to less than SG$10,000	1 (1.9%)	2 (8.7%)		4 (7.4%)	1 (7.1%)	
SG$10,000 and above	3 (5.8%)	0 (0.0%)		4 (7.4%)	5 (35.7%)	
Don’t know / Did not report	35 (67.3%)	12 (52.2%)		31 (57.4%)	5 (35.7%)	
Burden of kidney disease (k = 4)	66.1 (32.6)	58.2 (26.7)	0.31			
Symptoms/problems (k = 11)	82.6 (12.8)	85.8 (18.9)	0.40			
Effects of kidney disease (k = 8)	89.8 (10.3)	87.9 (13.6)	0.51			
SF-12 physical health composite	40.9 (10.2)	45.0 (8.6)	0.10			
SF-12 mental health composite	52.4 (10.8)	51.9 (8.8)	0.87			
Diagnosed with chronic conditions^b^						
Diabetes	32 (62.7%)	13 (56.5%)	0.61			
Cerebrovascular disease (e.g., stroke)	11 (21.6%)	0 (0.0%)	0.016			
Cardiovascular diseases	18 (34.6%)	7 (30.4%)	0.72			
Other conditions	9 (17.3%)	2 (8.7%)	0.33			
Cancer	10 (19.6%)	3 (13.0%)	0.49			
Relationship with patient? Patient is my…						0.42
Parent/Parent-in-law				38 (70.4%)	12 (85.7%)	
Spouse				12 (22.2%)	2 (14.3%)	
Other				4 (7.4%)	0 (0.0%)	

1 patient in the usual care arm and 1 caregiver in the intervention arm missed the postcounseling survey.

a ITE, Institute of Technical Education; JC, Junior college; Poly, polytechnic.

b Cardiovascular diseases include heart failure, heart attack, heart blood vessel narrowed or blocked, irregular heartbeat (dysrhythmia), other heart conditions (heart valve problems). Other conditions include chronic obstructive pulmonary disease, liver disease, gastrointestinal bleeding, poor circulation, or blocked blood vessels.

There were no deaths during the 6-month follow-up period. Three patients were lost to 6-month follow-up as no updated information could be identified for them in the electronic health records.

### Primary Outcome: Decisional Conflict

**Figure 1:**
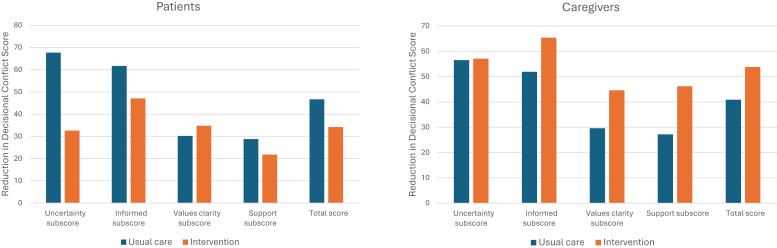
Reduction in decision conflict scale after counseling.

Patients in both study arms experienced reductions in decisional conflict following counseling. In the usual care arm, the average decision conflict score decreased from 56.63 precounseling to 9.90 postcounseling (mean change: -46.47, 95% CI: -52.33 to –40.62). In the intervention arm, the reduction went from 43.48 precounseling to 9.35 postcounseling (mean change: -34.13, 95% CI: -45.58 to -22.68) (Figure 1 and Supplementary Table S1). These changes show that the counseling results in a clinically meaningful improvement in decision conflict. However, there is no incremental improvement associated with the decision aid (β = -0.05; 95% CI: -2.57 to 2.47; *P* = 0.97) (Table 2).

**Table 2:** The Effect of intervention on decision conflict score

Change from pre− to post−counseling	Usual care		Intervention arm		Difference between arms			
	*n*	Mean (95% CI)	*n*	Mean (95% CI)	Unadjusted beta (95% CI)	*P*-value	Adjusted beta (95% CI)	*P*−value
Decisional conflict scale								
Total score								
Patients	51	−46.47 (−52.33 to −40.62)	23	−34.13 (−45.58 to −22.68)	−6.91 (−15.63 to 1.80)	0.120	−0.05 (−2.57 to 2.47)	0.969
Caregivers	54	−40.93 (−47.62 to −34.23)	13	−51.54 (−67.73 to −35.34)	−1.20 (−9.77 to 7.37)	0.784	−4.54 (−6.94 to −2.14)	< 0.001
Uncertainty subscore								
Patients	51	−67.16 (−77.91 to −56.40)	23	−32.61 (−46.60 to −18.61)	−8.74 (−20.07 to 2.60)	0.131	6.48 (1.81 to 11.15)	0.007
Caregivers	54	−56.48 (−68.13 to −44.84)	13	−53.85 (−75.95 to −31.74)	−4.70 (−16.18 to 6.77)	0.422	−2.83 (−6.54 to 0.89)	0.135
Informed subscore								
Patients	51	−61.11 (−70.88 to −51.34)	23	−47.10 (−61.46 to −32.74)	−12.04 (−21.41 to −2.68)	0.012	−1.88 (−5.56 to 1.79)	0.316
Caregivers	54	−51.85 (−60.31 to −43.39)	13	−64.10 (−80.47 to −47.74)	1.15 (−7.69 to 9.99)	0.799	−3.36 (−5.66 to −1.07)	0.004
Value subscore								
Patients	51	−30.88 (−42.53 to −19.24)	23	−34.78 (−49.98 to −19.59)	−1.11 (−11.49 to 9.27)	0.835	−3.72 (−8.14 to 0.71)	0.100
Caregivers	54	−29.63 (−40.24 to −19.02)	13	−40.39 (−62.22 to −18.55)	1.55 (−10.54 to 13.64)	0.802	−2.63 (−5.43 to 0.18)	0.067
Support subscore								
Patients	51	−28.43 (−35.47 to −21.4.)	23	−21.74 (−33.73 to −9.76)	−4.39 (−15.99 to 7.22)	0.459	−0.83 (−5.43 to 3.78)	0.725
Caregivers	54	−27.16 (−34.73 to −19.60)	13	−44.87 (−64.76 to −24.99)	−3.06 (−13.91 to 7.79)	0.581	−8.02 (−12.74 to −3.29)	0.001

CI, confidence interval.

Linear mixed model included fixed treatment effects and random intercepts. Adjusted models incorporated time and controlled for age, sex, education level, marital status, KDQOL-36 score (patient sample only), comorbidity status (patient sample only), and baseline measures.

For caregivers, the average decision conflict score decreased from 50.09 precounseling to 9.17 postcounseling (mean change: -40.93, 95% CI: -47.62 to -34.23) in the usual care arm versus from 55.00 to 1.15 (mean change: -51.54, 95% CI: -67.73 to -35.34) in the intervention arm. These improvements are again clinically and statistically significant. The decision aid was associated with an incremental reduction in the decision conflict score relative to counseling alone (β = -4.54; 95% CI: -6.94 to -2.14; *P* < 0.01), although the magnitude of the difference is small (Table 2). Pre- and post-counseling decisional conflict scores for each subscale are presented in Supplementary Table S1.

### Secondary Outcome: CM Awareness

Among patients, the increase in awareness of CM postcounseling was higher in the intervention arm (82.6%) compared with usual care (69.2%), with an adjusted OR of 7.24 (95% CI: 1.89–27.78; *P* < 0.01). Among caregivers, the increase in awareness was high in both groups (intervention: 71.4%; usual care: 77.8%), with those in the intervention arm having higher odds of increased awareness compared with those in the usual arm even after controlling for baseline awareness (OR = 4.50; 95% CI: 1.49–13.64; *P* = 0.01) (Table 3).

**Table 3:** Effect of the intervention on CM awareness

Change from pre- to post-counseling	Usual care		Intervention arm		Difference between arms			
	*n*	Proportion (95% CI)	*n*	Proportion (95% CI)	Unadjusted OR (95% CI)	*P*-value	Adjusted OR (95% CI)	*P*-value
Awareness of CM								
Patients	52	69.2% (52.7%–85.8%)	23	82.6% (62.8%–100%)	4.81 (1.69–13.70)	0.003	7.24 (1.89–27.78)	0.004
Caregivers	54	77.8% (63.7%–91.8%)	14	71.4% (40.6%–100%)	2.40 (0.74–7.83)	0.145	4.502 (1.49–13.64)	0.008

CI, confidence interval; CM, conservative management; OR, Odds ratio.

Mixed effects logistic model included fixed treatment effects and random intercepts. Adjusted models incorporated time and controlled for age, sex, education level, marital status, health status measured by Kidney Disease Quality of Life Instrument -36 (patient sample only), comorbidities status (patient sample only), and treatment awareness measured at baseline.

### Secondary Outcomes: Postcounseling Stated Treatment Preference and Actual Treatment Choice at 6 months

Patients in the intervention arm were less likely to prefer any type of dialysis after the counseling session compared with the usual care arm (OR = 0.33; 95% CI: 0.08–1.33; *P* = 0.12). Although this result was not statistically significant, it suggests a trend towards greater consideration of CM (Table 4). This trend is confirmed 6 months after counseling, as patients in the intervention arm were significantly less likely to have initiated any form of dialysis compared with those in the usual care arm (OR = 0.21; 95% CI: 0.043–0.99; *P* = 0.049) (Table 4).

**Table 4:** Effect of the intervention on treatment preferences

	Usual care	Intervention arm	Difference between arms			
	*n*	*n*	Unadjusted OR (95% CI)	*P*-value	Adjusted OR (95% CI)	*P-*value
Treatment preference post counseling: any form of dialysis						
Patients	51	23	0.42 (0.15–1.20)	0.106	0.33 (0.08–1.33)	0.118
Caregivers	54	13	0.69 (0.20–2.33)	0.546	0.61 (0.17–2.22)	0.451
Treatment preference post counseling: CM						
Patients	51	23	1.75 (0.64–4.80)	0.277	2.03 (0.61–6.77)	0.247
Caregivers	54	13	1.46 (0.43–4.96)	0.546	1.65 (0.45–6.01)	0.451
Initiated dialysis at 6 mo after counseling						
Patient	50	23	0.43 (0.15–1.25)	0.121	0.21 (0.04–0.99)	0.049

CI, confidence interval; CM, conservative management; OR, odds ratio.

Logistic regression model: results are presented in odds ratios. Adjusted models controlled for age, sex, education level, marital status, health status measured by Kidney Disease Quality of Life Instrument -36 (patient sample only), and comorbidity status (patient sample only).

## Discussion

This study evaluated the integration of a web-based PDA into kidney counseling sessions for older adults with ESKD and their caregivers, using a pragmatic pre- and post- trial design with a comparison arm. The PDA was developed to facilitate shared decision-making by presenting both dialysis and CM as viable treatment options, in contrast to standard educational materials, which primarily focus on dialysis.

Post counseling, the increase in CM awareness was higher in the intervention arm. This finding is not surprising given that standard educational materials did not mention CM as a treatment option. This highlights the role of PDAs in ensuring that patients and caregivers are fully informed about the spectrum of available treatments, which is essential for ethical and person-centered care.

Despite this increase in awareness, decision conflict did not significantly differ between patients who received the PDA and those in the usual care arm. Although conflict decreased post counseling in both arms, the magnitude of improvement was similar. One possible explanation is that the PDA introduced CM as an additional, viable option. Although this broadened patients’ understanding of their choices, it may also have added uncertainty as they contemplated 2 fundamentally different treatment pathways. In contrast, patients in the usual care arm may have focused primarily on selecting a dialysis modality, a narrower and more familiar decision, which could inherently feel less conflicting. Importantly, the added information in the intervention arm did not increase overall conflict. Instead, it may have encouraged more thoughtful and informed deliberation, even if it initially introduced more complexity into their decision-making process.

Caregivers, in contrast, reported significantly lower decisional conflict when the PDA was used. This may be because they are more cognitively and emotionally equipped to navigate the new information, being younger, healthier, and more educated than the patients. This finding reinforces the need to consider the different informational needs and processing capacities of patients and caregivers when designing and implementing decision support interventions. It also reinforces the potential of caregivers to help patients understand complicated medical information and make important medical decisions.

Patients in the intervention arm were also less likely to express a preference for dialysis over CM after the counseling session. Consistent with this result, a significantly lower proportion of patients in the intervention arm initiated dialysis 6 months after counseling as compared with those in usual care. It is likely that presenting patients with information about CM on an equal footing with dialysis, and including a values clarification exercise that helped patients better understand what is important to them, encouraged some patients to opt for CM who otherwise would not have. This underscores the value of the decision aid as an adjunct to the highly effective counseling sessions currently being offered. By explicitly presenting CM as a legitimate option, the PDA may have enabled patients to consider values such as maintaining quality of life, minimizing treatment burden, and aging in place—factors that are underrepresented in standard care discussions. This reveals the potential for the PDA to reduce overtreatment and better align care with best practices in geriatric nephrology, where individualized care planning is emphasized.

These results are broadly consistent with findings from other settings, though methodological differences warrant consideration. A United States randomized controlled trial of an interactive web-based decision aid for older adults with advanced CKD found reduced decisional conflict at 3 and 6 months, although effects diminished by 18 months.^39^ Interpretation of those results is limited, however, because many participants had not yet reached the point of making a dialysis decision, with enrollment including both stage 4 and stage 5 CKD. Nonetheless, both studies suggest that timely, balanced decision support may influence treatment choices and reduce decisional conflict, particularly when integrated into clinical care.

Our study has several limitations that should be noted. First, we evaluated the PDA as an add-on to standard counseling. As the counseling sessions themselves were highly effective at reducing decision conflict, the ability to show additional improvements from the PDA was limited. It is likely that in settings where standard counseling is non-existent or less thorough, the PDA would show much larger relative improvements. A key limitation of the study is that we were unable to reach the target sample size for the intervention arm before the project period ended. Recruitment was slower than anticipated, partly because of COVID-19–related restrictions that required us to pause recruitment. Even after the restrictions were lifted, many patients remained hesitant to spend additional time in clinical settings, which further limited participation. As a result, the smaller size of the intervention arm reduced the statistical power, limited the precision of effects estimates, and may have contributed to imbalance in baseline DCS scores between the study arms that may not be fully accounted for in the estimation. Future studies should consider employing adaptive recruitment approaches or interim sample size re-estimation strategies to ensure adequate power and more balanced group sizes. Another limitation is that we employed a pragmatic pre- and post- design with a comparison arm rather than a randomized controlled trial. This approach was selected for practical reasons, including limited recruitment capacity and concerns about contamination across study arms. However, because the control and intervention phases occurred sequentially rather than concurrently, secular trends or contextual changes, such as evolving counselor behavior, variations in patient mix, concurrent awareness campaigns, or the gradual recovery of clinical services following the COVID-19 period, may have contributed to observed differences. To overcome these limitations, future studies employing larger sample sizes and contemporaneous control groups should be conducted. Future research should also assess the scalability and long-term impact of such interventions across diverse healthcare settings.

This study adds to the growing body of suggestive evidence for the integration of PDAs into routine clinical. Although the primary outcome, patient decisional conflict, did not differ significantly between patient groups, the intervention produced several important findings. Patients exposed to the PDA demonstrated greater awareness of CM as a viable treatment option and were less likely to express a preference for, or subsequently initiate, dialysis. Notably, this study is among the few to demonstrate that counseling with a PDA can influence both treatment preferences and real-world treatment decisions. Caregivers who received the PDA reported significantly lower decisional conflict following counseling. Although the study was underpowered and employed a pre- and post-design with a comparison group, the results provide valuable insights into real-world implementation. By explicitly presenting CM as a legitimate treatment option, PDAs may enable patients and families to consider quality of life, treatment burden, and personal values when making decisions about dialysis initiation. Given the limitations, these findings should be considered only as suggestive evidence and confirmed in adequately powered randomized trials.

## Appendix

### List of members of the myKIDNEY Study Group

Jason Chon Jun Choo, Tazeen Jafar, Gek Cher Chan, Guozhang Lee, and Lydia Wei Wei Lim.

## Disclosure

All the authors declared no competing interests.

## Supplementary Material

Supplementary file (PDF) **Table S1.** Decision conflict scale scores pre- and post-counseling for the usual care arm and intervention arm. CONSORT 2025 Checklist.

## Footnotes

Supplementary file (PDF)
**Table S1.** Decision conflict scale scores pre- and post-counseling for the usual care arm and intervention arm. CONSORT 2025 Checklist.
